# Inhibition of MAP4K4 signaling initiates metabolic reprogramming to protect hepatocytes from lipotoxic damage

**DOI:** 10.1016/j.jlr.2022.100238

**Published:** 2022-06-06

**Authors:** Sumit Kumar Anand, Mara Caputo, Ying Xia, Emma Andersson, Emmelie Cansby, Sima Kumari, Marcus Henricsson, Rando Porosk, Katharina Susanne Keuenhof, Johanna Louise Höög, Syam Nair, Hanns-Ulrich Marschall, Matthias Blüher, Margit Mahlapuu

**Affiliations:** 1 Department of Chemistry and Molecular Biology, University of Gothenburg and Sahlgrenska University Hospital, Gothenburg, Sweden; 2 Biomarker Discovery and Development, Research and Early Development, Cardiovascular, Renal, and Metabolism (CVRM), BioPharmaceuticals R&D, AstraZeneca, Gothenburg, Sweden; 3 Department of Molecular and Clinical Medicine/Wallenberg Laboratory, Institute of Medicine, University of Gothenburg and Sahlgrenska University Hospital, Gothenburg, Sweden; 4 Department of Biochemistry, Institute of Biomedicine and Translational Medicine, University of Tartu, Tartu, Estonia; 5 Institute of Neuroscience and Physiology, and Institute of Clinical Sciences, Sahlgrenska Academy, University of Gothenburg, Gothenburg, Sweden; 6 Helmholtz Institute for Metabolic, Obesity, and Vascular Research (HI-MAG) of the Helmholtz Zentrum München, University of Leipzig and University Hospital Leipzig, Leipzig, Germany

**Keywords:** MAP4K4, non-alcoholic fatty liver disease, non-alcoholic steatohepatitis, liver steatosis, hepatic lipid metabolism, hepatic glycolysis, liver inflammation, oxidative/endoplasmic reticulum stress, hepatic fibrosis, 4-HNE, 4-hydroxynonenal, ACC, acetyl-CoA carboxylase, CHOP, C/EBP-homologous protein, DCFDA, dichlorodihydrofluorescein diacetate, ECAR, extracellular acidification rate, ER, endoplasmic reticulum, ERK, extracellular signal-regulated kinase, GFAP, glial fibrillary acidic protein, HCC, hepatocellular carcinoma, IHHs, immortalized human hepatocytes, JNK, c-Jun N-terminal kinase, MAP4K4, mitogen-activated protein kinase kinase kinase kinase 4, NAFLD, non-alcoholic fatty liver disease, NAS, NAFLD activity score, NASH, non-alcoholic steatohepatitis, NTC, non-targeting control, OCR, oxygen consumption rate, OMM, outer mitochondrial membrane, TAG, triacylglycerol, qRT-PCR, quantitative real-time PCR, si, small interfering, STE20, sterile 20

## Abstract

The primary hepatic consequence of obesity is non-alcoholic fatty liver disease (NAFLD), affecting about 25% of the global adult population. Non-alcoholic steatohepatitis (NASH) is a severe form of NAFLD characterized by liver lipid accumulation, inflammation, and hepatocyte ballooning, with a different degree of hepatic fibrosis. In the light of rapidly increasing prevalence of NAFLD and NASH, there is an urgent need for improved understanding of the molecular pathogenesis of these diseases. The aim of this study was to decipher the possible role of STE20-type kinase MAP4K4 in the regulation of hepatocellular lipotoxicity and susceptibility to NAFLD. We found that *MAP4K4* mRNA expression in human liver biopsies was positively correlated with key hallmarks of NAFLD (i.e., liver steatosis, lobular inflammation, hepatocellular ballooning, and fibrosis). We also found that the silencing of MAP4K4 suppressed lipid deposition in human hepatocytes by stimulating β-oxidation and triacylglycerol secretion, while attenuating fatty acid influx and lipid synthesis. Furthermore, downregulation of MAP4K4 markedly reduced the glycolysis rate and lowered incidences of oxidative/endoplasmic reticulum stress. In parallel, we observed suppressed JNK and ERK and increased AKT phosphorylation in MAP4K4-deficient hepatocytes. Together, these results provide the first experimental evidence supporting the potential involvement of STE20-type kinase MAP4K4 as a component of the hepatocellular lipotoxic milieu promoting NAFLD susceptibility.

Non-alcoholic fatty liver disease (NAFLD) is among the most prevalent metabolic disorders accompanying obesity, type 2 diabetes, and hyperlipidemia (1). Imbalance in liver fat metabolism leading to hepatic steatosis (defined as lipid accumulation in >5% of hepatocytes) is the first step in NAFLD (2). Subsequently, hepatocellular lipotoxicity fuels local inflammation and cell damage in the liver, driving the aggravation of NAFLD to non-alcoholic steatohepatitis (NASH), which in turn is the risk factor for the development of fibrosis, liver failure, and hepatocellular carcinoma (HCC) (3, 4). In spite of intensive research in this area of high unmet medical need, the molecular pathogenesis of NAFLD and NASH still remains elusive, and no approved drug is available for their treatment.

Proteins associating with intrahepatocellular lipid droplets are increasingly recognized as key regulators of liver lipid partitioning and NAFLD development/progression (5). Our recent studies have revealed that several sterile 20 (STE20)-type kinases—STK25, MST3, MST4, and TAOK3—decorate lipid droplets within hepatocytes to orchestrate intrahepatic fat accumulation as well as initiation and aggravation of NAFLD (6, 7, 8, 9, 10, 11, 12, 13, 14, 15). Interestingly, a genome-wide yeast two-hybrid (Y2H) screen of a primary human hepatocytes cDNA library identified mitogen-activated protein kinase kinase kinase kinase 4 [MAP4K4; also known as Nck-interacting kinase and hematopoietic progenitor kinase/germinal center kinase-like kinase] as an interaction partner of STK25 (15), which raised the hypothesis that MAP4K4 is also involved in the control of liver lipid homeostasis.

Similar to STK25, MAP4K4 is a member of the STE20 kinase superfamily. Original reports suggested a function for MAP4K4 upstream of the c-Jun N-terminal kinase (JNK) signaling cascade as a MAP4K (16, 17, 18, 19). Notably, single nucleotide polymorphisms in the *MAP4K4* locus have been reported to associate with insulin resistance and type 2 diabetes (20, 21). Mice with induced whole-body *Map4k4* depletion also display lower fasting blood glucose levels and improved systemic insulin sensitivity compared with control mice, when challenged with a high-fat diet (22). MAP4K4 expression and activity were found to be increased in the aortas of humans and mice with atherosclerosis (23). Moreover, treatment of *Apoe*^-/-^ and *Ldlr*^-/-^ mice with a selective small-molecule MAP4K4 inhibitor significantly reduces atherosclerotic lesion area, supporting the role of MAP4K4 as a potent regulator of vascular inflammation (23). Overexpression of MAP4K4 is detected in numerous human cancers (17, 24, 25). Consistently, studies in cell lines and animal models have revealed that MAP4K4 aggravates the migration and invasiveness of different types of cancer cells including breast, prostate, and ovarian cancer, malignant melanoma, glioblastoma, as well as HCC (17, 24, 26, 27, 28, 29).

In this study, we provide the first evidence to demonstrate that *MAP4K4* mRNA expression in human liver biopsies is positively correlated with NAFLD severity and that silencing of MAP4K4 in vitro protects human hepatocytes against lipotoxic damage.

## Materials and methods

### Analysis of hepatic *MAP4K4* mRNA expression from human participants

*MAP4K4* mRNA level was measured in liver biopsies obtained from 62 Caucasian individuals (men, n=35; women, n=27). The subjects were recruited among those undergoing laparoscopic abdominal surgery for Roux-en-Y bypass (n=12), sleeve gastrectomy (n=9), or elective cholecystectomy (n=41). The inclusion criteria were as follows: (1) men and women, age >18 years; (2) indication for elective laparoscopic or open abdominal surgery; (3) BMI between 18 to 50 kg/m^2^; (4) abdominal MRI feasible; and (5) signed written informed consent. The exclusion criteria were as follows: (1) significant acute or chronic inflammatory disease or clinical signs of infection; (2) CrP >10 mg/dl; (3) type 1 diabetes and/or antibodies against glutamic acid decarboxylase and islet cell antibodies; (4) systolic blood pressure >140 mmHg and diastolic blood pressure >95 mmHg; (5) clinical evidence of cardiovascular or peripheral artery disease; (6) thyroid dysfunction; (7) alcohol or drug abuse; and (8) pregnancy. Patients with type 2 diabetes (n=24) were diagnosed by a fasting plasma glucose >7.0 mmol/l and/or a 120 min oral glucose tolerance test glucose >11.1 mmol/l. For participant characteristics, see Cansby *et al.* (9).

Total body fat was assessed by dual X-ray absorptiometry. Hepatic fat content was analyzed by single-proton magnetic resonance spectroscopy as previously described (30). After food withdrawal overnight, a small liver biopsy was collected during the surgery (between 08:00 and 10:00 am), immediately snap frozen in liquid nitrogen, and stored at –80°C. NAFLD activity score (NAS) and fibrosis score were assessed on liver sections by a certified pathologist (31). Quantitative real-time PCR (qRT-PCR) was performed on liver biopsies as described below using the probes for MAP4K4 (Hs01101394_m1) and 18S rRNA (Hs99999901_s1; Thermo Fisher Scientific, Waltham, MA), which span exon-exon boundaries to improve the specificity.

All investigations were performed with approval by the Ethics Committee of the University of Leipzig, Germany (approval numbers 363-10-13122010 and 159-12-21052012) and carried out in accordance with the Declaration of Helsinki. Written informed consent to use their anonymized data was obtained from all patients enrolled in this study.

### Cell culture, RNA interference, and transient overexpression

Immortalized human hepatocytes (IHHs; a gift from B. Staels, the Pasteur Institute of Lille, University of Lille Nord de France, Lille, France) (32) were maintained in Complete William's E Medium (GlutaMAX supplemented; Gibco, Paisley, UK) supplemented with dexamethasone (50 nmol/l; Sigma-Aldrich, St. Louis, MO), human insulin (20 U/l; Actrapid Penfill; Novo Nordisk, Bagsværd, Denmark), 10% (vol/vol) FBS, and 1% (vol/vol) penicillin/streptomycin (Gibco). Cells were demonstrated to be free of mycoplasma infection by the MycoAlert Mycoplasma Detection Kit (Lonza, Basel, Switzerland).

IHHs were transfected with *MAP4K4* small interfering (si)RNA (Hs.701013; Ambion, Austin, TX) or scrambled siRNA (SIC001; Sigma-Aldrich) using Lipofectamine RNAiMax (Thermo Fisher Scientific). Cells were also transfected with human *MYC*-tagged *MAP4K4* expression plasmid (EX-A2245-M43; GeneCopoeia, Labomics, Nivelles, Belgium) or an empty control plasmid (EX-NEG-M43; GeneCopoeia) using Lipofectamine 2000 (Thermo Fisher Scientific). At 24 h posttransfections, the culture medium was replaced with fresh medium, with or without supplementation of 50 μmol/l oleic acid (Sigma-Aldrich), for subsequent 48 h incubation. In one experiment, MAP4K4-overexpressing cells were treated with a small-molecule MAP4K4 inhibitor PF-06260933 (Sigma-Aldrich (33)).

### Mice

Male mice of C57BL/6J strain (Charles River, Sulzfeld, Germany) were housed 3 to 5 per cage in a temperature-controlled (21°C) facility with a 12-h light-dark cycle and free access to chow and water. From the age of 6 weeks, mice were fed a high-fat diet (45 kcal% fat; D12451; Research Diets, New Brunswick, NJ) or a standard chow diet for 18 weeks. At the age of 24 weeks, the mice were euthanized, and liver samples were fixed in 4% (vol/vol) phosphate-buffered formaldehyde (Histolab Products, Gothenburg, Sweden), embedded in paraffin and sectioned or fixed in optimal cutting temperature mounting medium (Histolab Products) and frozen in liquid nitrogen, followed by cryosectioning. Liver tissues were also snap frozen in liquid nitrogen and stored at −80°C for protein measurement. The mice received human care according to the National Institutes of Health recommendations outlined in the Guide for the Care and Use of Laboratory Animals. All experiments were performed after prior approval from the local ethics committee (approval number 5.8.18–17,285/2018).

### Immunoelectron microscopy

Trypsinized IHHs were prepared in sealed pipette tips as previously described (34) but centrifuged at 500 g for 3 min. After transfer of the cells to aluminum carriers (M. Wohlwend, Sennwald, Switzerland), samples were high-pressure frozen, freeze-substituted, and sectioned as described (35), with the exception of the incubation in uranyl acetate lasting for 3 h. Immunolabeling and contrast staining were performed as in (36) using anti-MAP4K4 polyclonal antibody (1:30 dilution; Invitrogen, Carlsbad, CA) as primary antibody and goat anti-rabbit IgG (1:20 dilution; Electron Microscopy Services, Hatfield, PA) as secondary antibody with a 10 nm gold fiducial. Imaging was performed as in (35) with a pixel size of 0.453 nm for a magnification of 230,00X. Gold fiducials within 30 nm (approximate length of the antibody sandwich) of the outer mitochondrial membrane (OMM) were assigned to the OMM.

### Assessment of lipid metabolism and oxidative/endoplasmic reticulum stress

To quantify neutral lipids and superoxide radicals, IHHs were stained with 3.3 μg/ml Bodipy 493/503 (Invitrogen) at room temperature for 1 h or 5 μmol/l dihydroethidium (DHE; Life Technologies, Grand Island, NY) at 37°C for 5 min, respectively. Intracellular hydrogen peroxide (H_2_O_2_) was detected using the dichlorodihydro-fluorescein diacetate (DCFDA)/H2DCFDA-Cellular ROS Assay Kit according to the manufacturer’s instructions (Abcam, Cambridge, UK). Mitochondria and mitochondrial-derived superoxide were visualized by incubating IHHs with 50 nmol/l MitoTracker Deep Red (Thermo Fisher Scientific) or 5 μmol/l MitoSOX Red (Thermo Fisher Scientific), respectively, at 37°C for 20 min. In parallel, IHHs were processed for immunofluorescence with anti-8-oxoguanine (8-oxoG), anti-E06, anti-4-hydroxynonenal (4-HNE), anti-KDEL, anti-C/EBP-homologous protein (CHOP), anti-peroxisomal biogenesis factor 5 (PEX5), or anti-peroxisomal membrane protein 70 kDa (PMP70) antibodies (see supplemental Table S1 for antibody information). Immunofluorescence images were obtained using a Zeiss Axio Observer microscope with the ZEN Blue software (Zeiss, Oberkochen, Germany). The labeled area was assessed in 6 randomly selected 20X fields/well using the ImageJ software (1.47v; National Institutes of Health, Bethesda, MD). Oxidative damage to proteins was determined using the Protein Carbonyl Content Assay Kit (Sigma-Aldrich) following the manufacturer’s recommendations.

Mouse liver sections were stained with 3.3 μg/ml Bodipy 493/503 at room temperature for 1 h or 50 nmol/l MitoTracker Deep Red at 37°C for 20 min, and/or processed for immunofluorescence with anti-MAP4K4, anti-F4/80, or anti-glial fibrillary acidic protein (GFAP) antibodies (see supplemental Table S1 for antibody information).

Fatty acid uptake was assessed in IHHs using the Quencher-Based Technology Fatty Acid Uptake Assay Kit (Molecular Devices, Sunnyvale, CA). To measure triacylglycerol (TAG) secretion to the media, cells were first incubated with pulse media [Complete William’s E Medium containing 0.5 μCi/ml [^3^H]oleic acid (PerkinElmer, Waltham, MA), 360 μmol/l oleic acid (Sigma-Aldrich), and 1% (weight/vol) fatty acid-free BSA (Sigma-Aldrich)] for 8 h, followed by incubation with chase media [Complete William’s E Medium supplemented with 30% (weight/vol) fatty acid-free BSA] for 8 h. Media was collected for lipid extraction, followed by lipid separation by thin-layer chromatography on silica gel plates. Radiolabeled TAG was detected by iodine vapor and quantified using a scintillation counter (Tri-Carb 2800 TR; PerkinElmer). Incorporation of media-derived [^14^C]glucose and [^14^C]oleic acid into TAG was assessed by incubating IHHs with culture media containing 0.5 μCi/ml D-[^14^C]glucose (PerkinElmer), 10 μmol/l D-glucose (Sigma-Aldrich), and 1% (weight/vol) fatty acid-free BSA for 4 h or 0.5 μCi/ml [^14^C]oleic acid (PerkinElmer), 360 μmol/l oleic acid, and 1% (weight/vol) fatty acid-free BSA for 1.5 h, respectively. Cells were collected for the extraction of lipids, which were then separated by thin-layer chromatography. Radiolabeled TAG was detected by iodine vapor and quantified by scintillation counting. Glucose uptake into the cells was determined by adding 0.75 μCi/ml D-[^3^H]glucose (PerkinElmer) and 20 mmol/l 2-deoxy-D-glucose (Sigma-Aldrich) to the culture media. Glucose transport was stopped after 10 min by adding 20 mmol/l phloretin (Sigma-Aldrich). Cells were lysed in 0.2 mol/l NaOH and radioactivity was quantified in a scintillation counter. To measure β-oxidation, IHHs were incubated in culture media containing 0.5 μCi/ml [9,10-^3^H]palmitic acid (PerkinElmer), 110 nmol/ml palmitate (Sigma-Aldrich), and 1% (weight/vol) fatty acid-free BSA for 2 h and media was collected. The remaining labeled palmitate in the media samples was removed by adding 50 μl 20% (weight/vol) BSA and 27 μl 70% (vol/vol) perchloric acid followed by vortexing and centrifugation. The radioactive water formation as the product of free fatty acid oxidation was quantified in the supernatant using a liquid scintillation counter. Ketone body concentration and glucose production from pyruvate and lactate were measured in the culture medium using the Ketone Body Assay Kit (Sigma-Aldrich) and the Amplex Red Glucose/Glucose Oxidase Assay Kit (Invitrogen), respectively. Glycogen levels were assessed using the Glycogen Assay Kit (Sigma-Aldrich). Cell viability was analyzed using the CellTiter-Blue Cell Viability Assay (Promega, Stockholm, Sweden) according to the manufacturer’s protocol.

Membrane lipids were extracted using the BUME method (37); free cholesterol was measured using straight-phase HPLC with an evaporative light scattering detector (ELD) as previously described (38), while sphingomyelin, phosphatidylcholine, lysophosphatidylcholine, and phosphatidylethanolamine were quantified using direct infusion on a QTRAP 5500 Mass Spectrometer (Sciex, Concord, Canada) equipped with a robotic nanoflow ion source (TriVersa NanoMate; Advion Bio-Sciences, Ithaca, NJ) (39). Targeted metabolomics was carried out to analyze phosphohexoses and amino acids by multiple reaction monitoring scan on a QTRAP 4500 Mass Spectrometer (Sciex) as previously described (40).

### Seahorse assay

Cells were transfected in the Seahorse XF Cell Culture Microplate (Agilent Technologies, Santa Clara, CA) as described above. Real-time measurements of oxygen consumption rate (OCR) and extracellular acidification rate (ECAR) were performed on the Seahorse XFe96 Extracellular Flux Analyzer (Agilent Technologies). The XF Cell Mito Stress Test Kit (Agilent Technology) was used to determine the OCR as per the manufacturer’s protocol. In brief, the culture medium was replaced with the XF Base Medium supplemented with 10 mmol/l glucose, 1 mmol/l sodium pyruvate, and 2 mmol/l glutamine for 1 h. Oligomycin (4 μmol/l; ATP synthase inhibitor) was injected following basal OCR measurements, followed by injection of FCCP (2 μmol/l; a proton ionophore uncoupler inducing maximal respiration), and finally by injection of a mixture of rotenone (0.5 μmol/l; a complex I inhibitor) and antimycin A (0.5 μmol/l; a complex III inhibitor). For ECAR assessment, the culture medium was replaced with the Glycolysis Stress Assay Medium (Agilent Technologies) supplemented with 2 mmol/l glutamine for 1 h. Basal glycolysis rate was determined by injecting D-glucose (0.1 μmol/l). For determining glycolytic capacity, oligomycin was injected at a final concentration of 4 μmol/l. Finally, 2-deoxyglucose was injected at a final concentration of 0.5 μmol/l to measure the nonglycolytic acidification.

### qRT-PCR and Western blot

RNA was isolated from human liver biopsies and IHHs with the RNeasy Lipid Tissue Mini Kit (Qiagen, Hilden, Germany) and the EZNA Total RNA Kit (Omega Bio-Tek, Norcross, GA), respectively, according to the manufacturer’s instructions. cDNA was synthesized using the High-Capacity cDNA Reverse Transcription Kit (Thermo Fisher Scientific). Relative quantification was performed by the QuantStudio 6 Flex Real-Time PCR System (Thermo Fisher Scientific) or the CFX Connect Real-Time System (Bio-Rad, Hercules, CA). Relative quantities of target transcripts were assessed after normalization of the data to the endogenous control, 18S rRNA (Thermo Fisher Scientific). Western blot analysis was performed after measuring the protein concentrations using the BCA Protein Assay Kit (Thermo Fisher Scientific) to ensure equal loading of the protein. After the transfer of the proteins to the membrane (Trans-Blot Turbo Midi Nitrocellulose Transfer Membrane; Bio-Rad), the membranes were colored using 0.5% Ponceau (Merck Chemicals, Darmstadt, Germany) to verify that the transfer procedure had been successful. Specific proteins were detected using primary and secondary antibodies listed in supplemental Table S1.

### Statistical analysis

Statistical significance between the groups was investigated using the 2-sample Student's *t* test and among more than two groups by one-way ANOVA followed by a 2-sample Student's *t* test for post-hoc analysis. Differences were considered statistically significant at a *P* value <0.05. Correlation between *MAP4K4* expression in human liver biopsies and hepatic lipid content, NAS, and fibrosis score was evaluated by Spearman’s rank correlation analysis after the Kolmogorov–Smirnov test assessing normality of data. All statistical analyses were performed using SPSS statistics (v27; IBM Corporation, Armonk, NY).

## Results

### Hepatic *MAP4K4* expression is positively correlated with NAFLD severity

**Figure 1:**
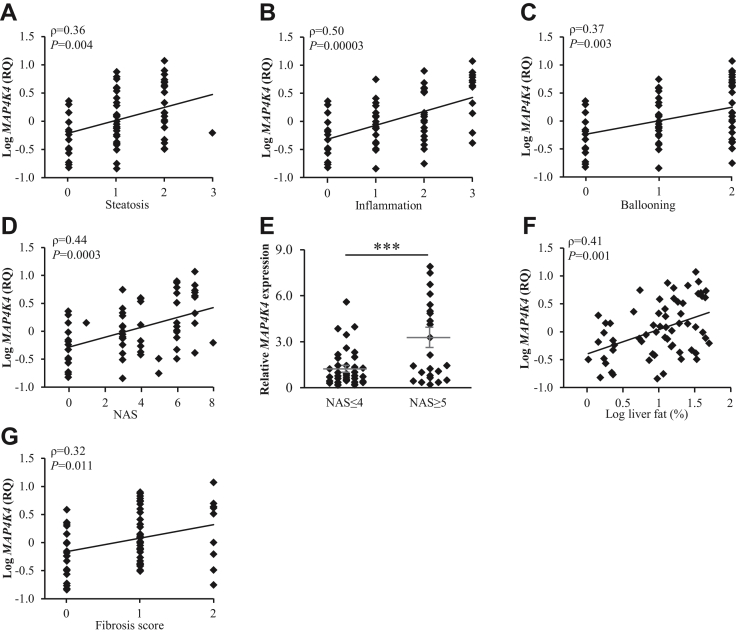
Hepatic *MAP4K4* expression is positively correlated with NAFLD severity. A–D: Correlation between *MAP4K4* transcript levels determined in human liver biopsies by qRT-PCR and the severity of the components of NAS (liver steatosis, inflammation, hepatocellular ballooning; A–C) as well as composite NAS (D). E: Hepatic *MAP4K4* expression in subjects with low versus high NAS (NAS ≤ 4 versus NAS ≥ 5, respectively). F–G: Correlation between hepatic *MAP4K4* mRNA abundance and liver fat assessed by magnetic resonance spectroscopy (F) as well as the histological score of liver fibrosis (G). Data are mean ± SEM. ∗∗∗*P* < 0.001. MAP4K4, mitogen-activated protein kinase kinase kinase kinase 4; NAFLD, non-alcoholic fatty liver disease; NAS, NAFLD activity score; RQ, relative quantification.

We analyzed the correlation between *MAP4K4* mRNA levels in human liver biopsies and the histological NAFLD severity score—NAS—in a cohort of 62 subjects (BMI: 22.7–45.6 kg/m^2^, body fat 19.5%–57.9%, liver fat 1.1%–50.0%). We detected a significant correlation between *MAP4K4* transcript abundance and the severity of the components of NAS (liver steatosis, inflammation, hepatocellular ballooning) as well as composite NAS (Fig. 1A–D). Notably, *MAP4K4* levels were 2.7 ± 0.4-fold higher in participants with NAS≥5 (defines definite NASH; n=24) compared with participants with NAS≤4 (defines simple steatosis or borderline NASH; n=38) (Fig. 1E). We also found a positive correlation between the hepatic *MAP4K4* expression and the liver fat assessed by magnetic resonance spectroscopy (Fig. 1F). Furthermore, the histological score of liver fibrosis was positively correlated with the hepatic *MAP4K4* expression (Fig. 1G). We observed no association between *MAP4K4* mRNA and the gender, BMI, body fat, waist-to-hip ratio, or HbA1c values of the subjects (supplemental Fig. S1).

Patients with NASH are at high risk of developing HCC, which is a poor-prognosis cancer type with a high recurrence rate (4). Notably, the analysis of the microarray data from the two HCC subject cohorts deposited at GEO database (n=214 for GSE14520 and n=91 for GSE102079) revealed that the *MAP4K4* gene expression was significantly higher in HCC tumors compared with adjacent non-tumor liver tissues (*P* < 0.0001).

### MAP4K4 is abundant in hepatocytes as well as liver non-parenchymal cells

**Figure 2:**
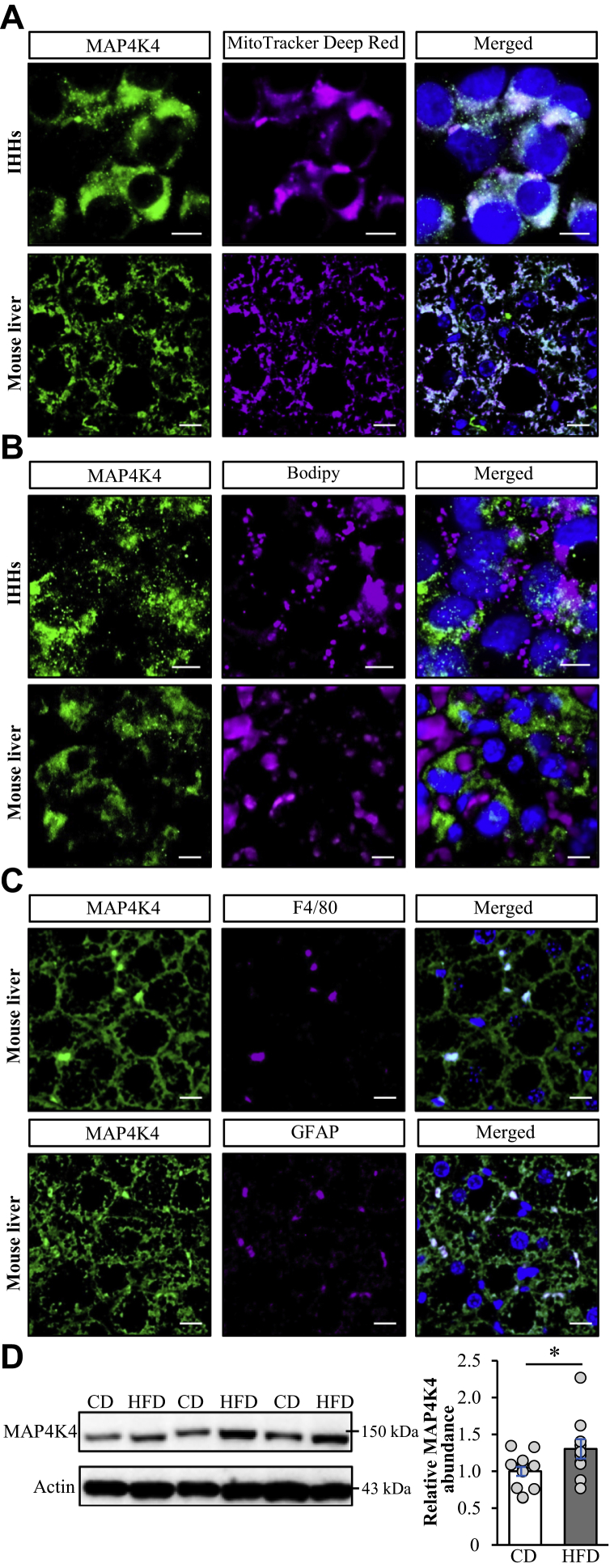
MAP4K4 is abundant in hepatocytes as well as liver non-parenchymal cells. A, B: Representative images of oleate-treated IHHs and high-fat diet–fed mouse liver sections, double-stained with anti-MAP4K4 (green) antibodies and MitoTracker Deep Red (violet; A) or Bodipy 493/503 (violet; B); colocalization is shown in white on the merged image; nuclei stained with DAPI (blue). The scale bars represent 10 μm. C: Representative images of high-fat diet–fed mouse liver sections double-stained with anti-MAP4K4 (green) and anti-F4/80 or anti-GFAP (violet) antibodies; colocalization is shown in white on the merged image; nuclei stained with DAPI (blue). The scale bars represent 10 μm. D: Liver lysates from high-fat diet–fed mice and age-matched controls fed a chow diet were analyzed by Western blot using anti-MAP4K4 antibodies. Densitometric analysis of protein levels and representative Western blots are presented (actin used as a loading control). For (D), data are mean ± SEM from 10-12 mice per group. ∗*P* < 0.05. CD, chow diet; HFD, high-fat diet; IHHs, immortalized human hepatocytes; MAP4K4, mitogen-activated protein kinase kinase kinase kinase 4.

MAP4K4 is widely expressed in human and rodent tissues including liver (22, 41). The subcellular localization of the protein appears to differ between cell types as MAP4K4 is reported to display diffuse cytoplasmic distribution pattern in NIH/373 mouse fibroblasts (42), while being concentrated at the tips of microtubule bundles in HaCaT human keratinocytes (43). Here, we examined the distribution of endogenous MAP4K4 in human hepatocytes and mouse liver. By wide-field immunofluorescence microscopy, we detected MAP4K4 protein in the cytoplasm of both human and mouse hepatocytes, where it largely colocalized with mitochondria, visualized by the staining with the fluorescent dye MitoTracker Deep Red (Fig. 2A); of note, we found no enrichment of MAP4K4 around intrahepatocellular lipid droplets (Fig. 2B). By using immunoelectron microscopy, we could detect MAP4K4 protein both in the mitochondrial matrix and close to the OMM in human hepatocytes (supplemental Fig. S2). Interestingly, MAP4K4 was highly expressed not only in mouse hepatocytes but also in liver non-parenchymal cells identified by immunostaining for F4/80 (macrophage marker) or GFAP [marker of hepatic stellate cells] (Fig. 2C). The protein abundance of MAP4K4 was slightly increased in the liver lysates from mice fed a high-fat diet compared with age-matched chow-fed mice (Fig. 2D).

### MAP4K4 controls lipotoxicity in human hepatocytes

**Figure 3:**
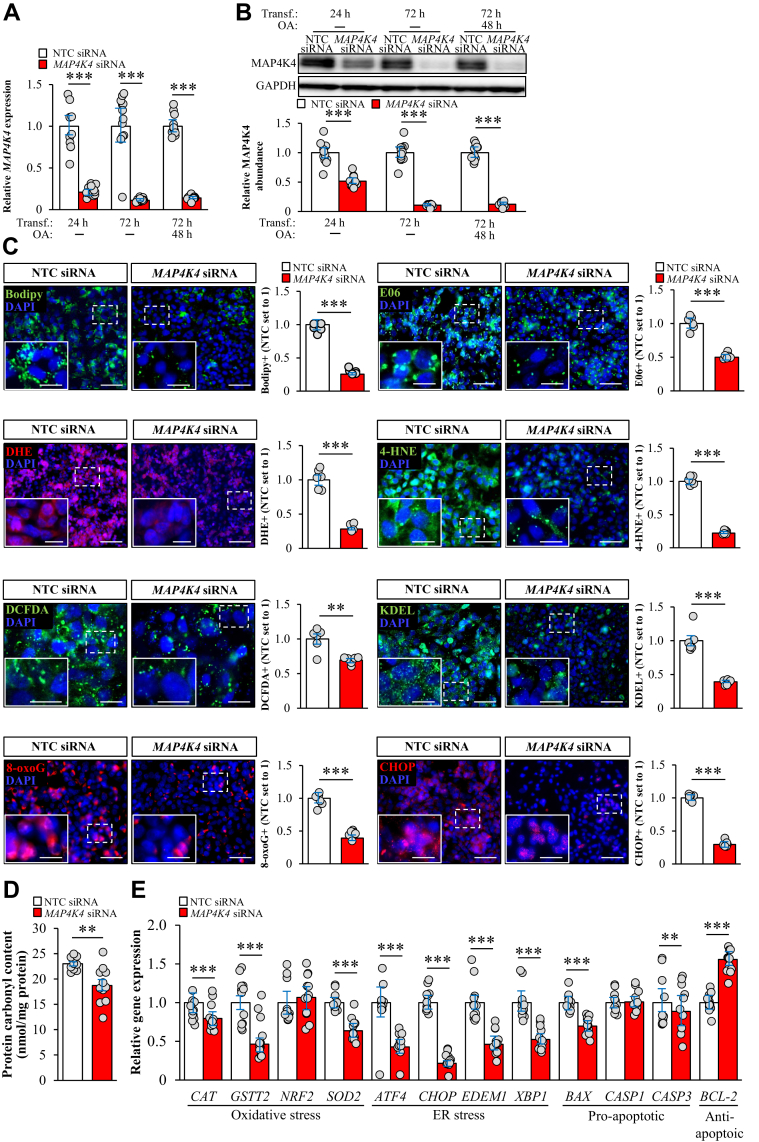
Silencing of MAP4K4 protects IHHs against lipotoxicity. IHHs were transfected with *MAP4K4* or NTC siRNA and incubated with oleic acid for 48 h. A, B: MAP4K4 mRNA (A) and protein (B) levels were analyzed by qRT-PCR and Western blot, respectively. In (B), densitometric analysis of protein levels and representative Western blots are presented [glyceraldehyde-3-phosphate dehydrogenase (GAPDH) used as a loading control]. C: Representative images of cells stained with Bodipy 493/503 (green), DHE (red), or DCFDA (green), or processed for immunofluorescence with anti-8-oxoG (red), anti-E06 (green), anti-4-HNE (green), anti-KDEL (green), or anti-CHOP (red) antibodies; nuclei stained with DAPI (blue). The scale bars represent 50 μm (20 μm in the zoomed view), except for the staining with DCFDA, where the scale bars represent 15 μm (10 μm in the zoomed view). Negative controls using nonimmune Ig instead of primary antibody did not show any positive staining (supplemental Fig. S13), verifying the specificity of the immunostaining. Quantification of the staining. D: Measurement of protein carbonylation levels. E: Relative mRNA levels of selected genes regulating oxidative/ER stress and apoptosis were analyzed by qRT-PCR. Data are mean ± SEM from 6 (C) or 12 (A, B, D, E) wells per group. ∗∗*P* < 0.01, ∗∗∗*P* < 0.001. OA, oleic acid; Transf., transfection. DHE, dihydroethidium; DCFDA, dichlorodihydrofluorescein diacetate; 8-oxoG, 8-oxoguanine; IHHs, immortalized human hepatocytes; MAP4K4, mitogen-activated protein kinase kinase kinase kinase 4; 4-HNE, 4-hydroxynonenal; 8-oxoG, 8-oxoguanine; CHOP, C/EBP-homologous protein.

To decipher the mechanism-of-action of MAP4K4 in NAFLD, we analyzed the effect of MAP4K4 knockdown on intracellular lipid accumulation as well as oxidative/endoplasmic reticulum (ER) stress in cultured human hepatocytes. We transfected IHHs with *MAP4K4*-specific siRNA or with a non-targeting control (NTC) siRNA; in parallel to the assays performed under basal cell culture conditions, we challenged the hepatocytes with oleic acid to mimic the environment in high-risk individuals. As expected, MAP4K4 mRNA and protein expression measured by qRT-PCR and Western blot, respectively, was efficiently silenced in IHHs transfected with *MAP4K4* siRNA (Fig. 3A, B). Consistently, immunostaining for MAP4K4 was substantially reduced in cells transfected with *MAP4K4* siRNA (supplemental Fig. S3).

First, we measured the amount of lipid droplets as a marker for lipotoxicity by quantifying the cell area stained with the lipophilic dye Bodipy 493/503. We detected about three-fold reduction in Bodipy-positive area in IHHs where MAP4K4 was silenced (Figs. 3C and S4A). Despite this marked decrease in cellular fat accumulation, MAP4K4 knockdown had no main impact on membrane lipid composition (supplemental Fig. S5). Interestingly, we found that MAP4K4-deficient IHHs were protected against oxidative stress as evidenced by lower abundance of intracellular superoxide radicals (O^2−^) and hydrogen peroxide (H_2_O_2_) assessed by DHE and DCFDA staining, respectively; decreased oxidative DNA and protein damage measured by immunostaining for IHHs were protected and by protein carbonylation assay, respectively; and declined deposition of oxidized phospholipids and lipid peroxidation products quantified by immunostaining for E06 and 4-HNE, respectively (Figs. 3C, D and S4A, B). We also observed suppressed immunostaining for KDEL (a signal motif for ER retrieval) and CHOP (an indicator of ER stress-induced cell death) in IHHs transfected with *MAP4K4* siRNA versus NTC siRNA (Figs. 3C and S4A). Consistently, the expression of several mRNA indicators of oxidative and ER stress as well as proapoptotic markers was significantly reduced in MAP4K4-deficient IHHs, in parallel with increased transcript levels of the antiapoptotic protein BCL-2 (Figs. 3E and S4C). Importantly, the impact of MAP4K4 knockdown was similar in hepatocytes cultured with or without oleate supplementation, except for H_2_O_2_ abundance, which was decreased by MAP4K4 silencing only in IHHs treated with oleic acid (Figs. 3C and S4A).

**Figure 4:**
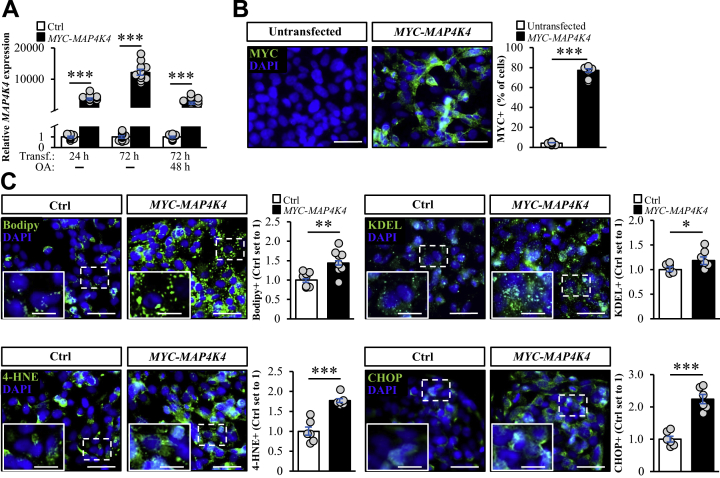
Overexpression of MAP4K4 aggravates lipotoxicity in IHHs. IHHs were transfected with *MYC*-tagged *MAP4K4* expression plasmid or an empty vector and incubated with oleic acid for 48 h A: *MAP4K4* mRNA abundance was assessed by qRT-PCR. B: Representative images of cells processed for immunofluorescence with anti-MYC (green) antibodies; nuclei stained with DAPI (blue). The scale bars represent 25 μm. Quantification of the MYC-positive cells (%). C: Representative images of cells stained with Bodipy 493/503 (green) or processed for immunofluorescence with anti-4-HNE, anti-KDEL, or anti-CHOP (green) antibodies; nuclei stained with DAPI (blue). The scale bars represent 25 μm (10 μm in the zoomed view). Quantification of the staining. Data are mean ± SEM from 6-8 (B, C) or 12 (A) wells per group. ∗*P* < 0.05, ∗∗*P* < 0.01, ∗∗∗*P* < 0.001. Ctrl, empty control plasmid; OA, oleic acid; Transf., transfection; IHHs, immortalized human hepatocytes; MAP4K4, mitogen-activated protein kinase kinase kinase kinase 4; 4-HNE, 4-hydroxynonenal; CHOP, C/EBP-homologous protein.

In parallel to the silencing of MAP4K4, we also overexpressed this kinase in IHHs, to examine whether an increased MAP4K4 abundance would lead to a reciprocal effect on hepatocellular lipotoxicity compared with MAP4K4 knockdown. IHHs transfected with human *MYC*-tagged *MAP4K4* expression plasmid displayed substantially higher *MAP4K4* levels compared with cells transfected with an empty control plasmid, with a high transfection efficacy (about 80% of cells displayed immunostaining with anti-MYC antibodies; Fig. 4A, B). In contrast to our findings in MAP4K4-deficient cells, we detected exacerbated intrahepatocellular lipid storage as well as oxidative and ER stress in IHHs overexpressing MAP4K4 as evidenced by a significant increase in the area labeled with Bodipy 493/503, 4-HNE, and KDEL/CHOP, respectively, which was observed both with and without oleate supplementation in the culture medium (Figs. 4C and S6). Furthermore, the treatment of oleate-loaded IHHs transfected with *MYC-MAP4K4* with a highly selective small-molecule MAP4K4 inhibitor PF-06260933 (described in detail elsewhere (33)) significantly suppressed lipid content and oxidative/ER stress (supplemental Fig. S7).

Importantly, reduced or increased abundance of MAP4K4 had no effect on the viability of IHHs (supplemental Fig. S8).

### Hepatocellular MAP4K4 silencing induces a shift from lipid anabolism to catabolism and reduces the glycolysis rate

**Figure 5:**
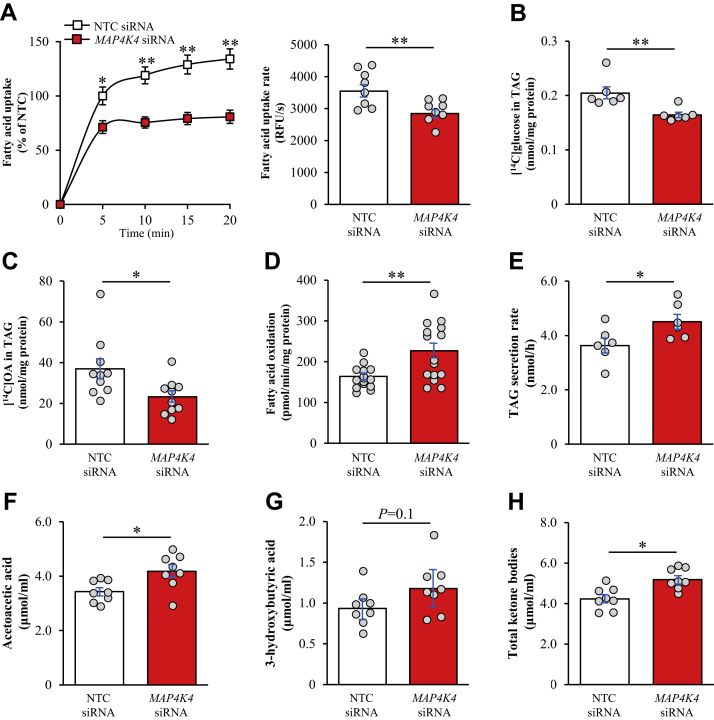
Knockdown of MAP4K4 in IHHs suppresses fatty acid uptake and lipid synthesis and promotes β-oxidation and TAG secretion. IHHs were transfected with *MAP4K4* or NTC siRNA and incubated with oleic acid for 48 h. A: Fatty acid uptake rate. B, C: TAG synthesis from [^14^C]-labeled glucose (B) and [^14^C]-labeled oleic acid (C). D: Oxidation of radiolabeled palmitate. E: Secretion of [^3^H]TAG into the medium. F–H: Ketone body concentration in the culture medium. Data are mean ± SEM from 6-10 (A–C and E–H) or 15 (D) wells per group. ∗*P* < 0.05, ∗∗*P* < 0.01. IHHs, immortalized human hepatocytes; MAP4K4, mitogen-activated protein kinase kinase kinase kinase 4; NTC, non-targeting control; OA, oleic acid.

Next, we investigated the mechanisms behind the reduced lipid storage induced by the silencing of MAP4K4 in human hepatocytes. We found that fatty acid influx and TAG synthesis from glucose as well as oleic acid (i.e., lipid anabolism) were significantly suppressed in MAP4K4-deficient IHHs exposed to oleic acid (Fig. 5A–C). Reciprocally, MAP4K4 knockdown in oleic acid-treated IHHs resulted in augmented β-oxidation measured by quantification [^3^H]-labeled water as the product of [9,10-^3^H(N)]palmitic acid oxidation as well as a higher secretion of de novo synthesized TAG into the media (i.e., lipid catabolism; Fig. 5D, E). In line with enhanced β-oxidation, we detected increased production of ketone bodies in oleate-loaded IHHs transfected with *MAP4K4* siRNA versus NTC siRNA (Fig. 5F, H). Notably, we found that the protein abundance of acetyl-CoA carboxylase (ACC) was lower in MAP4K4-deficient IHHs whereas the ratio of phospho-ACC (inactive form)/ACC was not changed (supplemental Fig. S9A). This provides a possible mode-of-action for MAP4K4 to regulate lipid synthesis and utilization since the enzymatic product of ACC, malonyl-CoA, is a precursor of fatty acid synthesis, and it also suppresses β-oxidation through allosteric inhibition of mitochondrial carnitine palmitoyltransferase (44). Furthermore, we found that the mRNA levels of steatogenic transcription factors *Pparg* and *Srebp1* were significantly lower, while the expression of *Pgc1a* known to activate hepatic fatty acid oxidation and ketogenesis (45) was higher, in IHHs where MAP4K4 was depleted (supplemental Fig. S9B).

**Figure 6:**
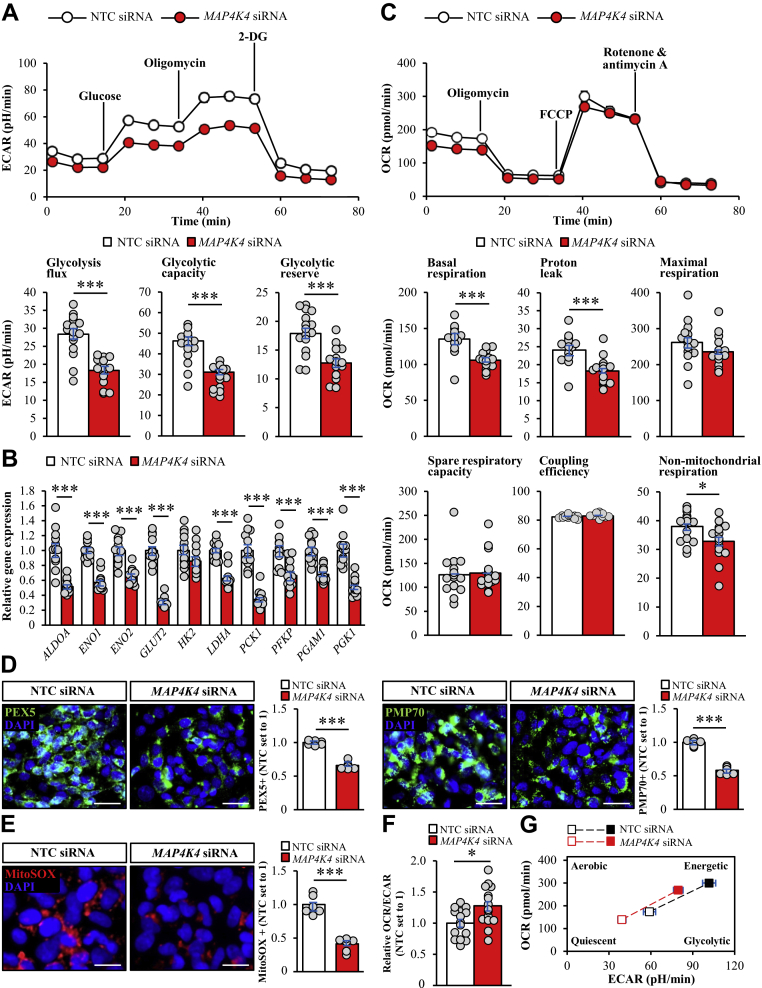
MAP4K4 silencing in IHHs induces a shift from glycolytic to mitochondrial pathways. IHHs were transfected with *MAP4K4* or NTC siRNA and incubated with oleic acid for 48 h. A, C: Measurement of ECAR (A) and OCR (C) using the Seahorse XFe96 Extracellular Flux Analyzer. B: Relative mRNA expression of selected genes controlling glycolysis was quantified by qRT-PCR. D–E: Representative images of cells processed for immunofluorescence with anti-PEX5 or anti-PMP70 (green) antibodies (D) or stained with MitoSOX (red; E); nuclei stained with DAPI (blue). The scale bars represent 25 μm. Quantification of the staining. F: The ratio of OCR to ECAR indicating hepatocellular preference for oxidative phosphorylation versus glycolysis. G: The ECAR and OCR values were used to generate cell energy phenotype profiles by the XF Cell Energy Phenotype Report Generator. The baseline activity (open symbols) and the metabolic activity in response to stressors (closed symbols) are connected by dashed lines. Data are mean ± SEM from 6 (D, E) or 12–16 (A–C and F–G) wells per group. ∗*P* < 0.05, ∗∗∗*P* < 0.001. IHHs, immortalized human hepatocytes; MAP4K4, mitogen-activated protein kinase kinase kinase kinase 4; NTC, non-targeting control; PEX5, peroxisomal biogenesis factor 5; PMP70, peroxisomal membrane protein 70 kD; ECAR, extracellular acidification rate; OCR, oxygen consumption rate.

We also measured ECAR representing glycolysis and OCR representing mitochondrial oxidative phosphorylation in oleate-laden IHHs transfected with *MAP4K4* siRNA versus NTC siRNA using the Seahorse XF Analyzer. When subjected to a glycolysis stress test, attenuation in the steady-state glycolysis flux and glycolytic capacity was observed in IHHs where MAP4K4 was silenced (Fig. 6A). The glycolytic reserve, which indicates the capability of a cell to respond to the glycolysis demand, was also decreased in IHHs where MAP4K4 was depleted (Fig. 6A). Consistently, the transcript levels of several glycolytic enzymes were significantly reduced by MAP4K4 depletion (Fig. 6B). Interestingly, the markedly lower glycolysis rate in MAP4K4-deficient IHHs was not accompanied by any decline in glucose uptake (supplemental Fig. S10A). Furthermore, we did not detect any differences in glycogen content or gluconeogenesis rate in IHHs transfected with *MAP4K4* siRNA versus NTC siRNA (supplemental Fig. S10B, C).

In a mitochondrial stress test, we detected lower basal OCR and reduced proton leak across mitochondrial membrane in IHHs transfected with *MAP4K4* siRNA versus NTC siRNA without any alterations in maximal respiration, spare respiratory capacity, or coupling efficiency (Fig. 6C). Non-mitochondrial respiration was decreased in IHHs where MAP4K4 was knocked down (Fig. 6C), and consistently, the peroxisomal activity was alleviated in MAP4K4-deficient cells as evidenced by reduced immunostaining for peroxisome biogenesis marker PEX5 and peroxisomal membrane protein PMP70 (Fig. 6D). We also found that MAP4K4 depletion in IHHs markedly suppressed the intramitochondrial ROS production measured by the staining of cells with the mitochondrially targeted fluorescent superoxide indicator MitoSOX Red (Fig. 6E). Importantly, the silencing of MAP4K4 increased the basal OCR/ECAR ratio in hepatocytes (Fig. 6F), suggesting a metabolic shift from relative utilization of glycolytic to mitochondrial pathways for energy production. In parallel, we detected a small reduction in the utilization of both glycolytic and aerobic pathways in IHHs transfected with *MAP4K4* siRNA versus NTC siRNA, both under basal and stressed conditions (Fig. 6G).

Targeted metabolomics analysis revealed that the concentration of phosphohexoses as well as several amino acids was significantly higher in MAP4K4-deficient IHHs (supplemental Fig. S11), supporting a shift in energy metabolism from the use of carbohydrates and amino acids toward lipids.

### Changes in extracellular signal ERK, JNK, and AKT signaling accompany MAP4K4-dependent lipotoxicity in hepatocytes

**Figure 7:**
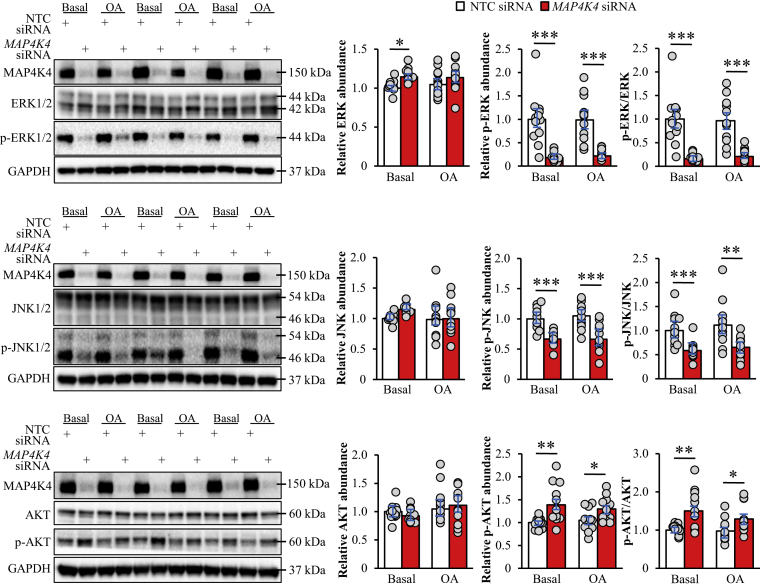
MAP4K4 affects ERK, JNK, and AKT activation in IHHs. IHHs were transfected with *MAP4K4* or NTC siRNA and cultured with and without oleate supplementation. Cell lysates were assessed by Western blot using antibodies for ERK1/2, phospho-ERK (Thr^202^/Tyr^204^), JNK1/2/3, phospho-JNK (Thr^183^/Tyr^185^), AKT, phospho-AKT (Ser^473^), or MAP4K4. Densitometric analysis of protein levels and representative Western blots are presented (GAPDH used as a loading control). Data are mean ± SEM from 11-12 wells per group. ∗*P* < 0.05, ∗∗*P* < 0.01, ∗∗∗*P* < 0.001. IHHs, immortalized human hepatocytes; MAP4K4, mitogen-activated protein kinase kinase kinase kinase 4; NTC, non-targeting control; OA, oleic acid; ERK, extracellular signal-regulated kinase; JNK, c-Jun N-terminal kinase.

To further decipher the mechanisms by which MAP4K4 silencing protects hepatocytes against lipotoxic damage, we performed immunoblot analysis in IHHs transfected *MAP4K4* siRNA versus NTC siRNA to monitor the activation status of mitogen-activated protein kinases including ERK and JNK, which are important signaling components controlling hepatocellular lipid content and development of steatohepatitis (46, 47, 48). We found that the phosphorylation of ERK (at Thr^202^/Tyr^204^) and JNK (at Thr^183^/Tyr^185^) was significantly lower in IHHs where MAP4K4 was knocked down, both under basal culture conditions and after exposing cells to oleic acid (Fig. 7). Notably, increased AKT activation has been reported in the livers from inducible whole-body *Map4k4* knockout mice (22). Consistently, we detected elevated phosphorylation of AKT in MAP4K4-deficient IHHs cultured with or without oleate supplementation (Fig. 7).

## Disussion

**Figure 8:**
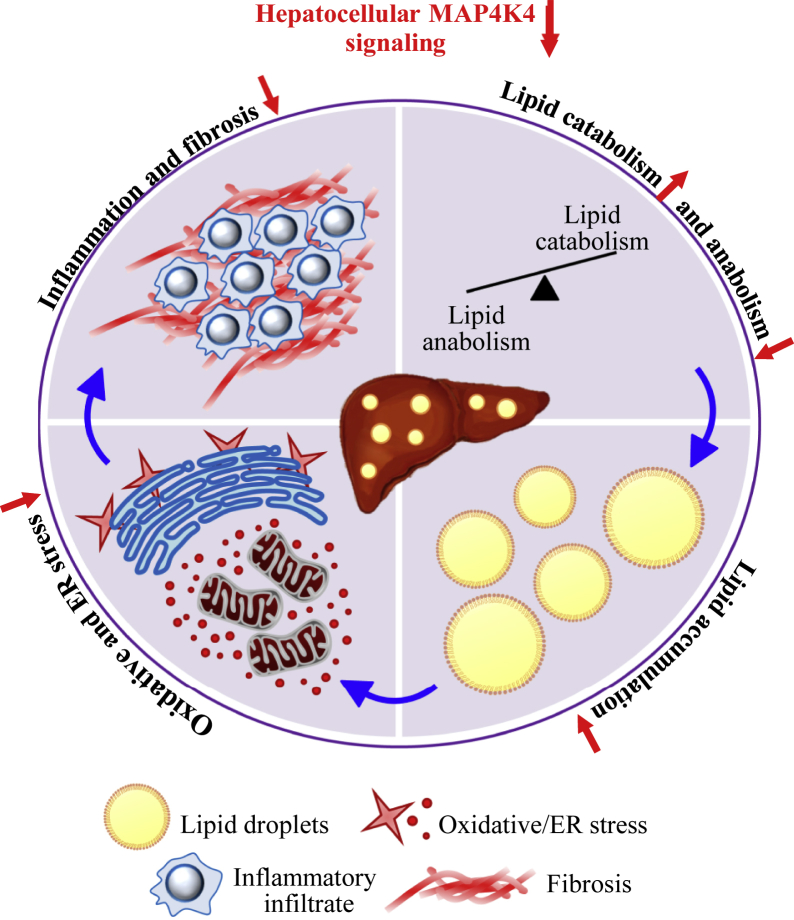
A schematic model describing the function of MAP4K4 in the regulation of hepatocellular lipotoxicity and NAFLD susceptibility. Silencing of MAP4K4 in hepatocytes stimulates lipid droplet catabolism and inhibits lipid droplet anabolism, protecting the cells against oxidative and ER stress. Although not examined in this study, these changes in MAP4K4-deficient hepatocytes are expected to lower the susceptibility of the liver to inflammation and fibrotic damage. MAP4K4, mitogen-activated protein kinase kinase kinase kinase 4; NAFLD, non-alcoholic fatty liver disease.

Hepatocellular lipotoxicity plays a pivotal role in the molecular pathogenesis of NAFLD and also triggers its advanced stage NASH by fueling local inflammation and fibrosis (49, 50). However, the exact mechanisms underlying initial lipid-induced liver damage, and NAFLD to NASH progression, have yet to be fully elucidated. In the present study, we provide the first evidence for the role of STE20-type kinase MAP4K4 in exacerbating liver lipotoxicity (Fig. 8). We demonstrate that the silencing of MAP4K4 protected human hepatocytes against lipid accumulation as well as oxidative/ER stress and, reciprocally, MAP4K4 overexpression rendered hepatocytes more susceptible to fat deposition and metabolic stress. Furthermore, we found that *MAP4K4* levels in human liver biopsies were positively correlated with the key lesions of NAFLD/NASH (i.e., hepatic steatosis, inflammation, fibrosis, and hepatocellular ballooning).

Interestingly, we have previously identified MAP4K4 as an interaction partner of another STE20-type kinase STK25 in human hepatocytes (15). The possibility that MAP4K4 and STK25 act in the same signaling pathway was strengthened by the results of this study demonstrating that the impact of MAP4K4 silencing on intrahepatocellular lipid metabolism and oxidative/ER stress is similar to the earlier reported effect of STK25 knockdown (7, 8, 14). Notably, while the subcellular localization of STK25 in human and mouse hepatocytes is confined to lipid droplets (6, 8, 9, 14), MAP4K4 protein was largely colocalized with mitochondria without any enrichment detected around intrahepatocellular lipid droplets. Importantly, lipid droplets in hepatocytes are known to physically interact with a variety of membrane-bound cellular organelles including the mitochondria, both in dynamic and stable setting, and the lipid droplet-mitochondria association may be further stabilized via fusion of the outer membrane leaflets (5, 51, 52, 53). It is therefore possible that MAP4K4 and STK25 proteins interact at the contact sites between mitochondria and lipid droplets. However, we surmise that, at this juncture, we are only in the infancy of understanding the molecular composition and regulation of mitochondria–lipid droplet tethering, and this complex matter requires further studies.

Mechanistically, this study reveals that the inhibition of MAP4K4 signaling effectively protected human hepatocytes against “fat overload” by suppressing fatty acid influx and TAG synthesis (i.e., input) and stimulating β-oxidation and TAG efflux (i.e*.*, output). We also found that the downregulation of MAP4K4 markedly reduced the glycolysis rate, assessed by the measurement of glucose to lactate conversion, in human hepatocytes. The alteration from aerobic oxidation to lactic acid fermentation even in oxygen-rich conditions is termed as “Warburg effect” and is one of the pivotal metabolic shifts in the process of carcinogenesis, providing tumor cells with several proliferative and survival advantages (54). Thus, our study provides an important insight into the previously reported involvement of MAP4K4 in promoting the HCC malignant phenotype (24, 27, 28).

Remarkably, we found that the silencing of MAP4K4 in hepatocytes activated several features of hepatic starvation response, including stimulation of fatty acid β-oxidation and ketogenesis, decrease in glycolysis and lipogenesis, and increase in *Pgc1a* expression as well as AKT phosphorylation. However, enhanced glycogenolysis and gluconeogenesis, which also characterize the transition from fed to fasted states in the liver, were not observed in MAP4K4-deficient hepatocytes. Of note, we also detected a significantly lower abundance of endogenous MAP4K4 protein in IHHs and HepG2 cells after starvation (supplemental Fig. S12). Together, these results suggest that MAP4K4 antagonism could trigger some but not all aspects of hepatic fasting response.

MAP4K4 has been described as an upstream activator of the JNK cascade in some (17, 55, 56, 57) but not all cell types (23, 58, 59, 60). The role of MAP4K4 in ERK signaling also appears to be cell type specific; for example, MAP4K4 phosphorylates ERK in skeletal muscle cells isolated from human biopsies (61) but not in rodent C2C12 myoblasts (59). Notably, the possible impact of MAP4K4 on the regulation of hepatic JNK or ERK signaling has not been studied previously. To this end, we here found that the silencing of MAP4K4 markedly lowered both JNK and ERK phosphorylation level in human hepatocytes. This observation is interesting in the light of recent reports revealing that activation of hepatic JNK and ERK pathways mediates NAFLD initiation and aggravation; however, the underlying mechanisms have remained largely elusive (46, 47, 62, 63, 64). In all, it is plausible that alterations in JNK and ERK signaling may constitute an important part of the mode-of-action of MAP4K4 in the control of NAFLD susceptibility.

We acknowledge that all the in vitro experiments in this study were carried out using IHHs, which may not fully represent the metabolic profile of hepatocytes in vivo. Thus, additional in vivo investigations as well as analyses in human primary hepatocytes are necessary to further characterize the impact of MAP4K4 silencing on liver lipid and glucose metabolism.

In conclusion, our data obtained by combining experiments using gene silencing and pharmacological inhibitors in immortalized human hepatocytes with expression profiling in human liver biopsies support the potential role of STE20-type kinase MAP4K4 as a component of the hepatocellular lipotoxic milieu. Further studies in animal models are warranted to examine whether antagonizing hepatic MAP4K4 signaling may be beneficial in NAFLD as well as other metabolic diseases, where liver lipotoxicity is being the principal pathological mechanism.

## Data availability

All data generated and/or analyzed during the study are presented in this article and are available from the corresponding author upon reasonable request.

## Supplemental data

This article contains supplemental data.

## Conflicts of interest

The authors declare that they have no conflicts of interest with the contents of this article.
