# Supplementary material for: Inhibition of MAP4K4 signaling initiates metabolic reprogramming to protect hepatocytes from lipotoxic damage
Source: J Lipid Res. 2022 Jun 6;63(7):100238. doi: 10.1016/j.jlr.2022.100238 (PMC9293639; doi:10.1016/j.jlr.2022.100238)
Supplement: Supplemental Table 1 [file mmc1.docx]

**Supplemental Table S1.** List of antibodies used for Western blot and immunofluorescence analysis

| **Type** | **Antibody name and catalogue number** | **Working dilution** | **Company** |  |
| --- | --- | --- | --- | --- |
| Primary | anti-MAP4K4 (#3485) | 1:1000 | Cell Signaling Technology (Boston, MA) | |
| antibody | anti-MAP4K4 (PA5-104232) | 1:500 | Invitrogen (Carlsbad, CA) | |
|  | anti-F4/80 (MCA497GA) | 1:250 | Bio-Rad (Hercules, CA) | |
|  | anti-GFAP (13-0300) | 1:500 | Invitrogen | |
|  | anti-Actin (#A2920) | 1:500 | Santa Cruz Biotechnology (Santa Cruz, CA) | |
|  | anti-GAPDH (sc-47724) | 1:1000 | Santa Cruz Biotechnology | |
|  | anti-8-oxoG (ab62623)  anti-E06 (J0914) | 1:500  1:100 | Abcam (Cambridge, UK)  Avanti Polar Lipids, Inc. (Alabaster, AL) | |
|  | anti-4-HNE (ab46545) | 1:500 | Abcam | |
|  | anti-KDEL (ab176333)  anti-CHOP (MA1-250)  anti-MYC (PA1-981)  anti-PEX5 (PA5-58716)  anti-PMP70 (PA1-650) | 1:500  1:200  1:100  1:500  1:500 | Abcam  Invitrogen  Invitrogen  Invitrogen  Invitrogen | |
|  | anti-ERK1/2 (#9102)  anti-p-ERK1/2 (#9101) | 1:1000  1:1000 | Cell Signaling Technology  Cell Signaling Technology | |
|  | anti-JNK1/2 (#9252) | 1:1000 | Cell Signaling Technology | |
|  | anti-p-JNK1/2 (#4668) | 1:1000 | Cell Signaling Technology | |
|  | anti-AKT (#9272) | 1:1000 | Cell Signaling Technology | |
|  | anti-p-AKT (#9271)  anti-ACC (#3662)  anti-p-ACC (#3661) | 1:1000  1:1000  1:1000 | Cell Signaling Technology  Cell Signaling Technology  Cell Signaling Technology | |
| Secondary antibody | Alexa Fluor-488-labeled anti-mouse IgG (A21202) | 1:500 | Invitrogen | |
|  | Alexa Fluor-488-labeled anti-rabbit IgG (A11008) | 1:500 | Invitrogen | |
|  | Alexa Fluor-594-labeled anti-mouse IgG (A11005) | 1:500 | Invitrogen | |
|  | Alexa Fluor-594-labeled anti-rabbit IgG (A21207) | 1:500 | Invitrogen | |
|  | anti-rabbit IgG (#7074) | 1:1000 | Cell Signaling Technology | |
|  | anti-mouse IgG (#7076) | 1:1000 | Cell Signaling Technology | |
