# Supplementary material for: Inhibition of MAP4K4 signaling initiates metabolic reprogramming to protect hepatocytes from lipotoxic damage
Source: J Lipid Res. 2022 Jun 6;63(7):100238. doi: 10.1016/j.jlr.2022.100238 (PMC9293639; doi:10.1016/j.jlr.2022.100238)
Supplement: Supplemental Table 2 [file mmc2.docx]

**Supplemental Table S2.** Specificity of the labeling (A) and localization of the label within mitochondria (B) of immuno-electron microscopy analysis in IHHs using anti-MAP4K4 antibody. IHHs were cultured under basal conditions or exposed to oleic acid for 48 hours. At least 100 mitochondria were imaged per condition.

| **Oleic acid**  **Primary antibody** |  | **+** | **+** | **+**  **+** |
| --- | --- | --- | --- | --- |
| Total number of mitochondria | 111 | 106 | 114 | 107 |
| Number of  mitochondria  with fiducials | 1 | 0 | 18 | 9 |
| Number of  fiducials | 2 | 0 | 23 | 11 |
| % of mitochondria  with gold labeling | 0.9 | 0.0 | 15.8 | 8.4 |

**B**

**A**

| **Oleic acid**  **Primary antibody** | **+** | | | **+**  **+** | |
| --- | --- | --- | --- | --- | --- |
| Number of  mitochondria | 45 | | | 30 | |
| Number of  fiducials | 42 | | | 38 | |
| % of fiducials  detected within  30 nm of OMM | 19 | | | 18 | |
|  | |  |  | |  |
