# Supplementary material for: Inhibition of MAP4K4 signaling initiates metabolic reprogramming to protect hepatocytes from lipotoxic damage
Source: J Lipid Res. 2022 Jun 6;63(7):100238. doi: 10.1016/j.jlr.2022.100238 (PMC9293639; doi:10.1016/j.jlr.2022.100238)
Supplement: Supporting Figure [file mmc3.pdf]

Supplementary Figure 1

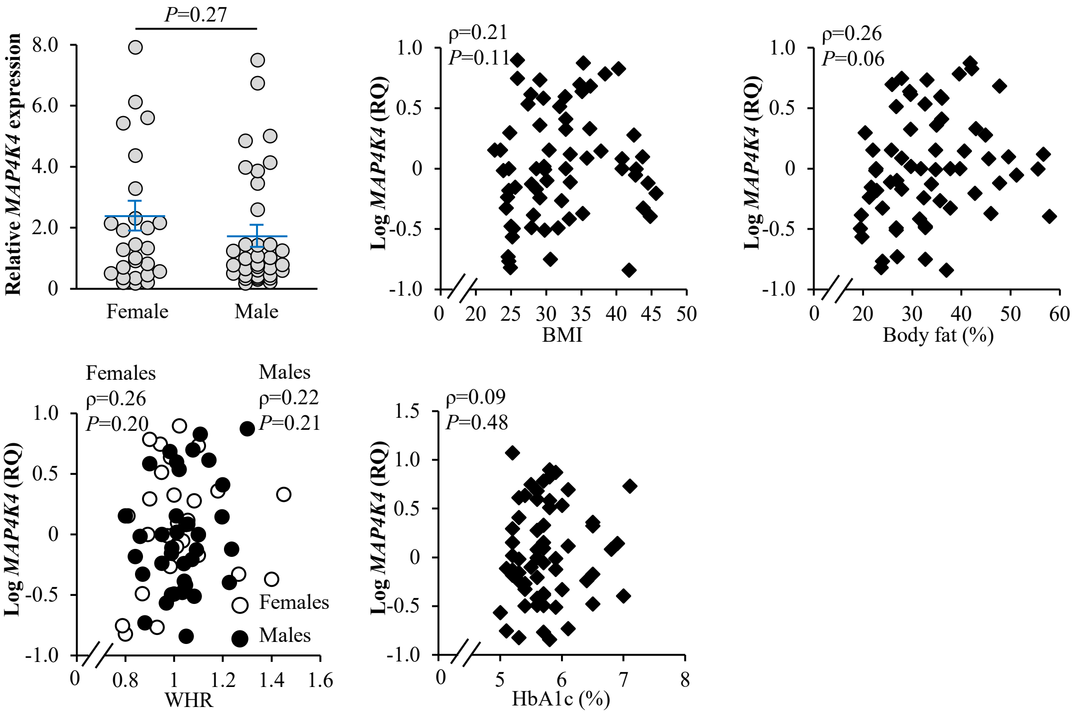

Supplementary Figure 1. No correlation is observed between the hepatic expression of *MAP4K4* mRNA and gender, BMI, body fat content, WHR, or HbA1c values of the subjects. *MAP4K4* mRNA expression was quantified in liver biopsies by qRT-PCR. RQ, relative quantification

Supplementary Figure 2

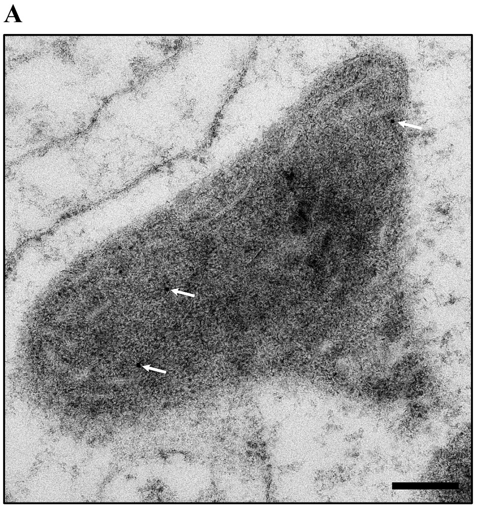

Supplementary Figure 2. MAP4K4 protein presents both in the mitochondrial matrix and OMM in human hepatocytes. (A-B) Representative immuno-electron microscopy images of IHHs cultured under basal conditions (A) or exposed to oleic acid for 48 hours (B). Antibody specificity was ascertained by imaging samples excluding the primary antibody (Supplementary Table 2). Arrows indicate gold fiducials. The scale bars represent 200 nm.

Supplementary Figure 3

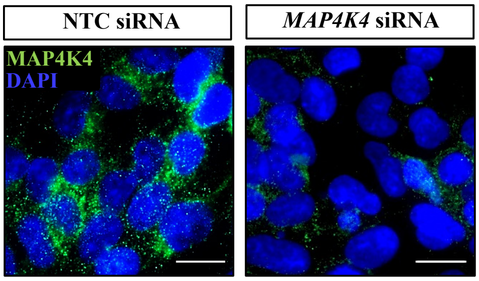

Supplementary Figure 3. Immunostaining for MAP4K4 is substantially reduced in cells transfected with *MAP4K4* siRNA. IHHs were transfected with *MAP4K4* siRNA or NTC siRNA and challenged with oleic acid for 48 hours. Representative images of cells processed for immunofluorescence with anti-MAP4K4 (green) antibodies; nuclei stained with DAPI (blue). The scale bars represent 25 µm.

Supplementary Figure 4

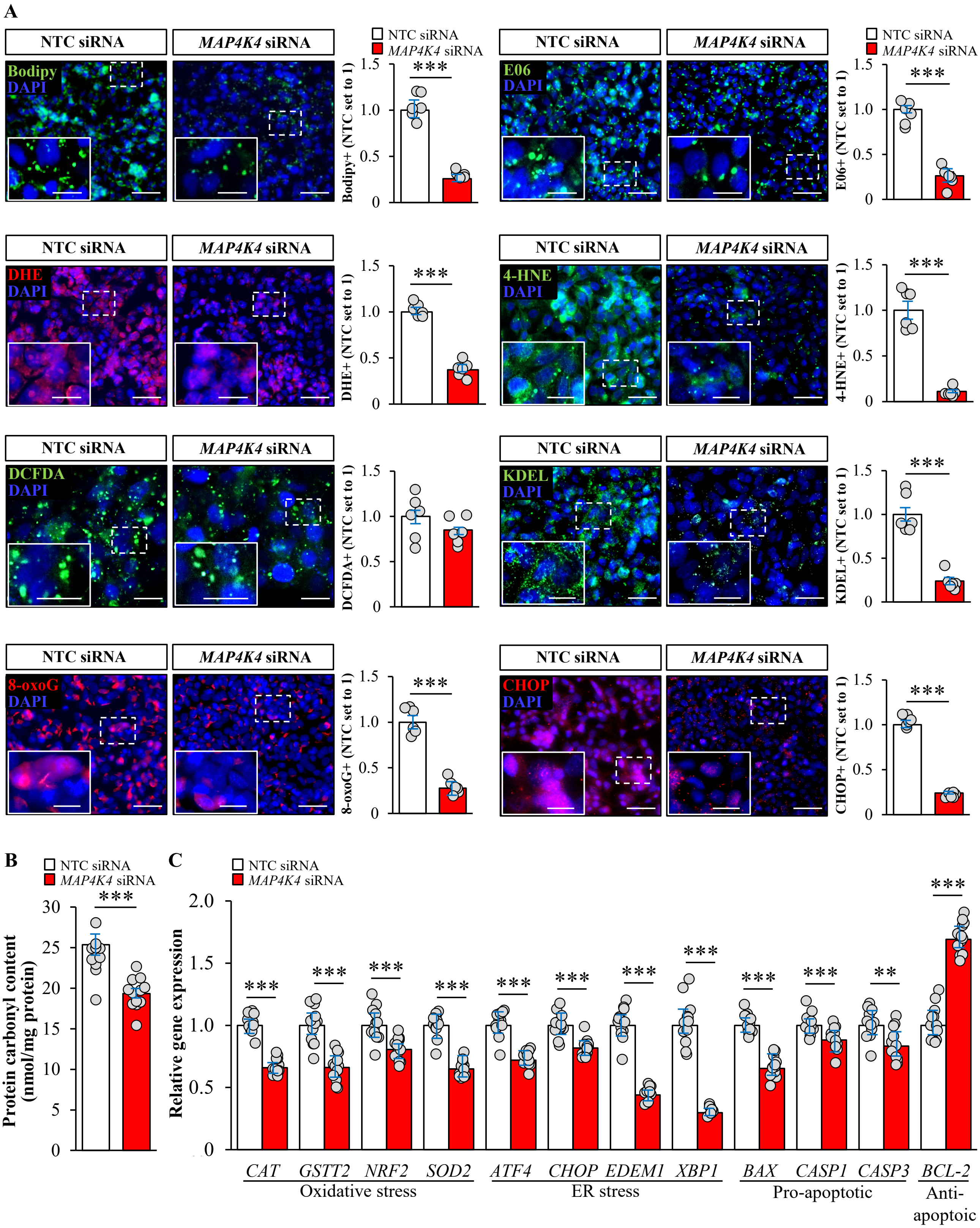

Supplementary Figure 4. Silencing of MAP4K4 protects IHHs against lipotoxicity. IHHs were transfected with *MAP4K4* siRNA or NTC siRNA and cultured under basal conditions. (A) Representative images of cells stained with Bodipy 493/503 (green), DHE (red), or DCFDA (green), or processed for immunofluorescence with anti-8-oxoG (red), anti-E06 (green), anti-4-HNE (green), anti-KDEL (green), or anti-CHOP (red) antibodies; nuclei stained with DAPI (blue). The scale bars represent 50 µm (20 µm in the zoomed view), except for the staining with DCFDA, where the scale bars represent 15 µm (10 µm in the zoomed view). Quantification of the staining. (B) Measurement of protein carbonylation levels. (C) Relative mRNA expression of selected genes controlling oxidative and ER stress as well as apoptosis was assessed by qRT-PCR. Data are mean ± SEM from 6 (A) or 12 (B-C) wells per group. $^{**}P<0.01$, $^{***}P<0.001$

Supplementary Figure 5

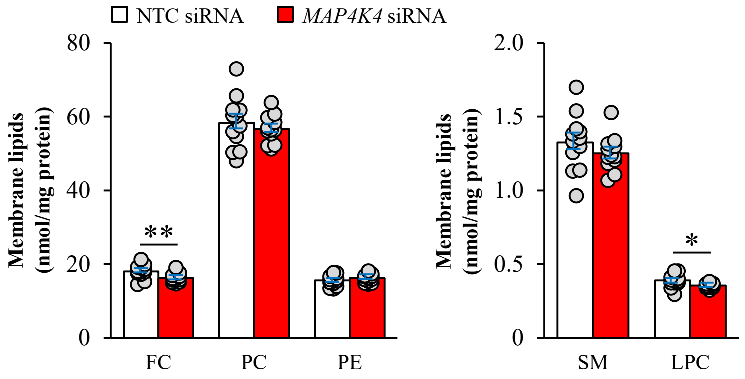

Supplementary Figure 5. Knockdown of MAP4K4 in IHHs has no major impact on the composition of membrane lipids. IHHs were transfected with *MAP4K4* siRNA or NTC siRNA and challenged with oleic acid for 48 hours. Lipidomic analysis was performed after extraction of membrane lipids. Data are mean ± SEM from 12 wells per group. FC, free cholesterol; LPC, lysophosphatidylcholine; PC, phosphatidylcholine; PE, phosphatidylethanolamine; SM, sphingomyelin. *P<0.05, **P<0.01

Supplementary Figure 6

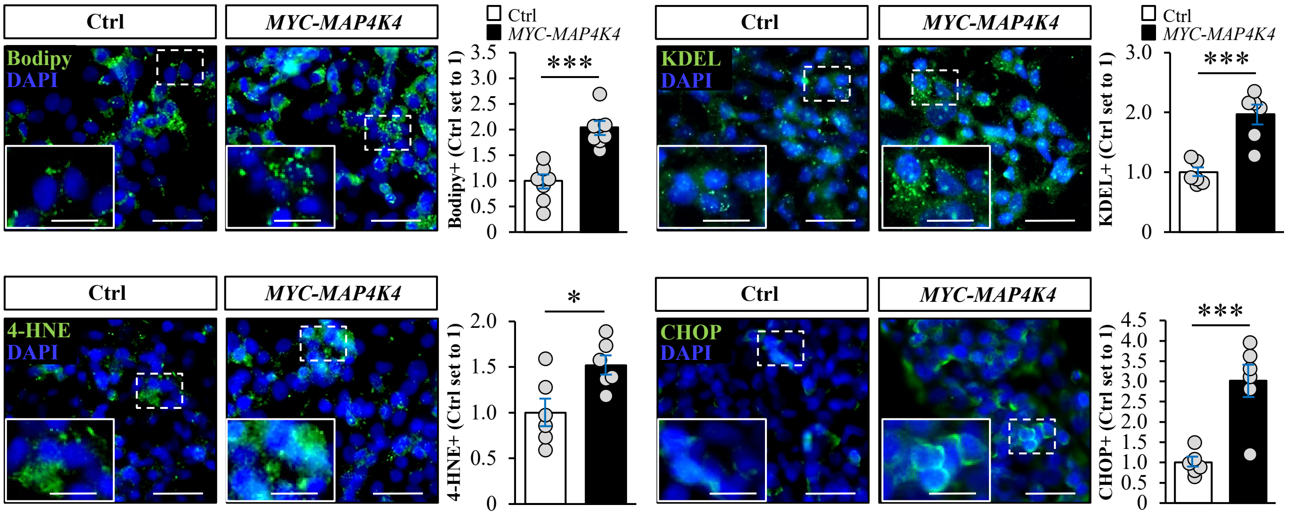

Supplementary Figure 6. Overexpression of MAP4K4 aggravates lipotoxicity in IHHs. IHHs were transfected with *MYC*-tagged *MAP4K4* expression plasmid or an empty control plasmid and cultured under basal conditions. Representative images of cells stained with Bodipy 493/503 (green) or processed for immunofluorescence with anti-4-HNE, anti-KDEL, or anti-CHOP (green) antibodies; nuclei stained with DAPI (blue). The scale bars represent 25 µm (10 µm in the zoomed view). Quantification of the staining. Data are mean ± SEM from 6-8 wells per group. Ctrl, empty control plasmid. *P<0.05, ***P<0.001

Supplementary Figure 7

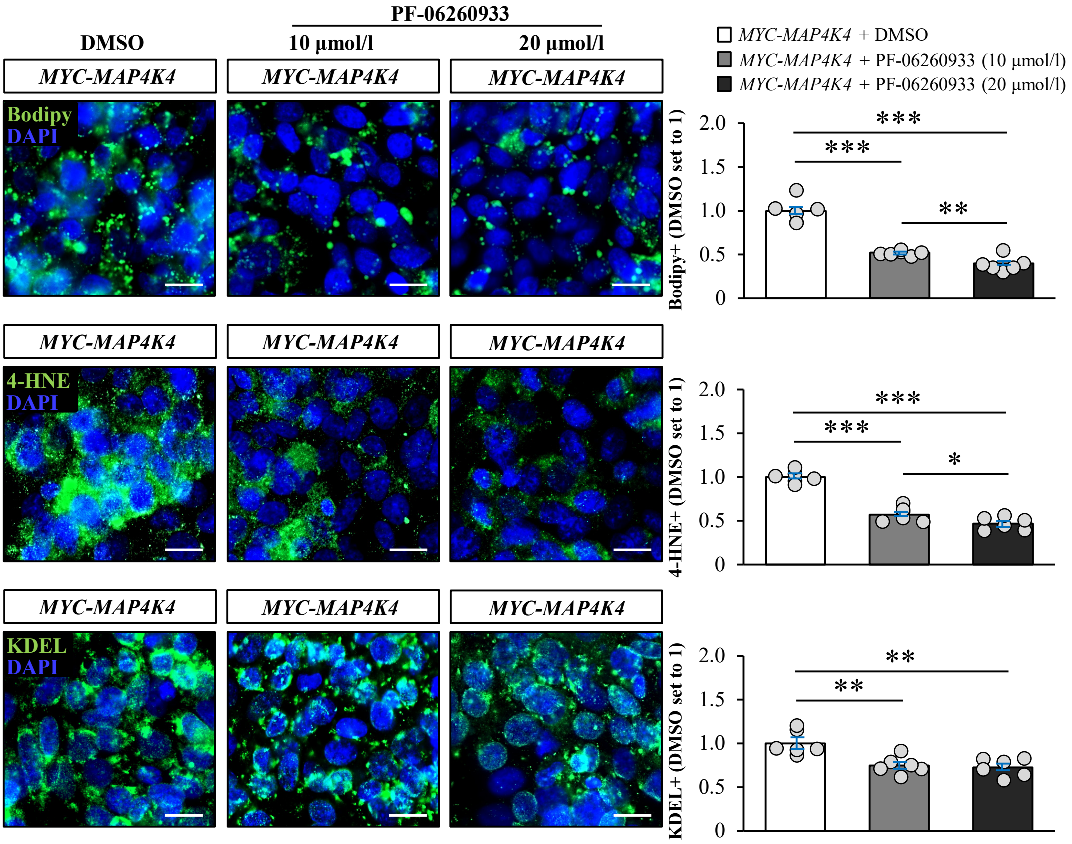

Supplementary Figure 7. Pharmacological MAP4K4 inhibition reduces lipid accumulation, oxidative, and ER stress in IHHs. IHHs were transfected with *MYC*-tagged *MAP4K4* expression plasmid, challenged with oleic acid for 48 hours, and incubated with PF-06260933 (a small-molecule inhibitor of MAP4K4) for 2 hours. Representative images of cells stained with Bodipy 493/503 (green), or processed for immunofluorescence with anti-4-HNE or anti-KDEL (green) antibodies; nuclei stained with DAPI (blue). The scale bars represent 10 µm. Quantification of the staining. Data are mean ± SEM from 6 wells per group. DMSO, dimethyl sulfoxide. *P<0.05, **P<0.01, ***P<0.001

Supplementary Figure 8

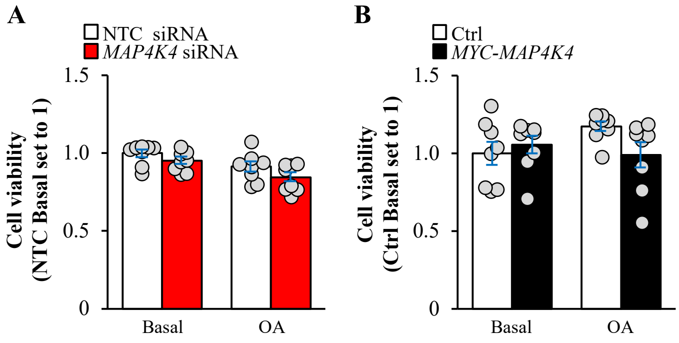

Supplementary Figure 8. Modifying the abundance of MAP4K4 in IHHs has no impact on cell viability. (A-B) IHHs were transfected with *MAP4K4* siRNA or NTC siRNA (A) or with *MYC*-tagged *MAP4K4* expression plasmid or an empty control plasmid (B). The assessments were performed under basal culture conditions and after exposing cells to oleic acid for 48 hours. Data are mean ± SEM from 8 wells per group. Ctrl, empty control plasmid; OA, oleic acid

Supplementary Figure 9

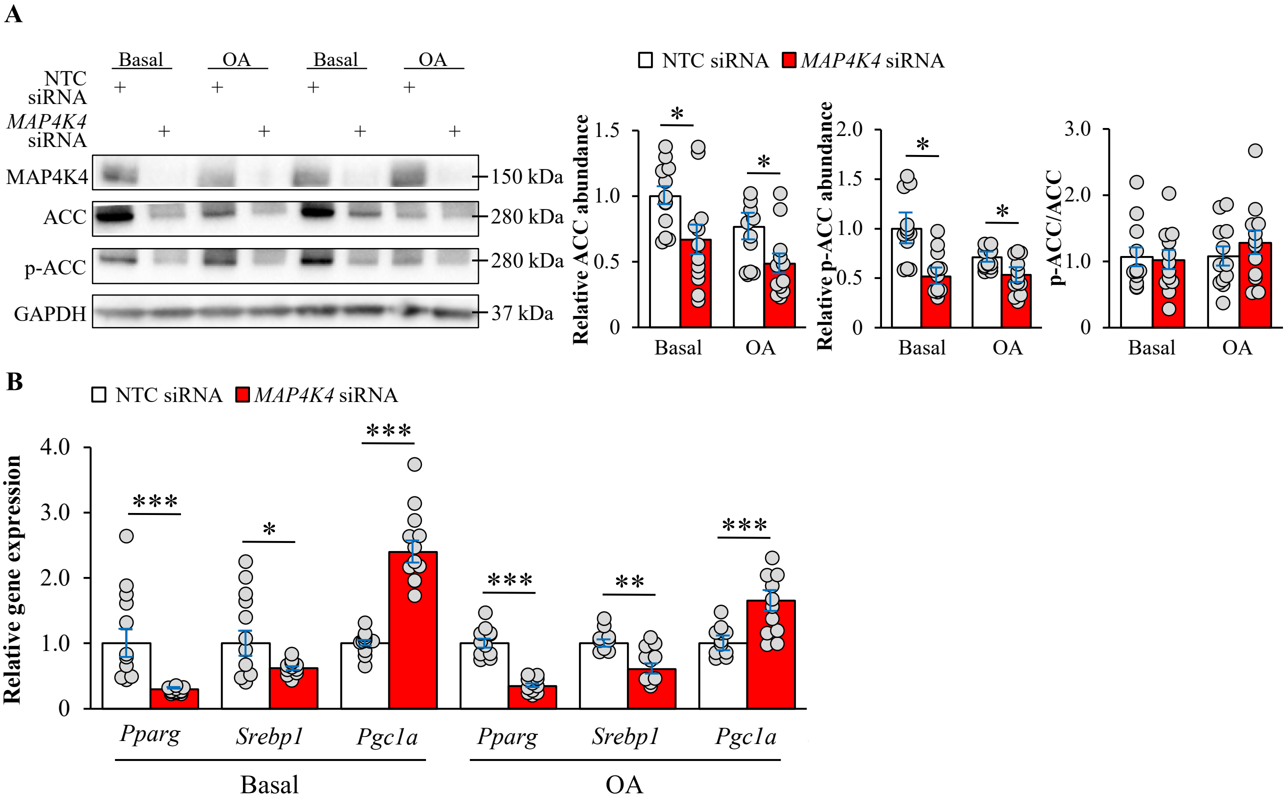

Supplementary Figure 9. Knockdown of MAP4K4 in IHHs regulates the protein abundance of ACC and the transcript levels of several transcription factors. IHHs were transfected with *MAP4K4* siRNA or NTC siRNA, and cultured with and without oleate supplementation. (A) Cell lysates were assessed by Western blot using antibodies for ACC, phospho-ACC (Ser^{79}), or MAP4K4. Densitometric analysis of protein levels and representative Western blots are presented (GAPDH used as a loading control). (B) Relative mRNA expression of selected transcriptional regulators of lipid metabolism was quantified by qRT-PCR. Data are mean ± SEM from 11-12 wells per group. OA, oleic acid. *P<0.05, **P<0.01, ***P<0.001

Supplementary Figure 10

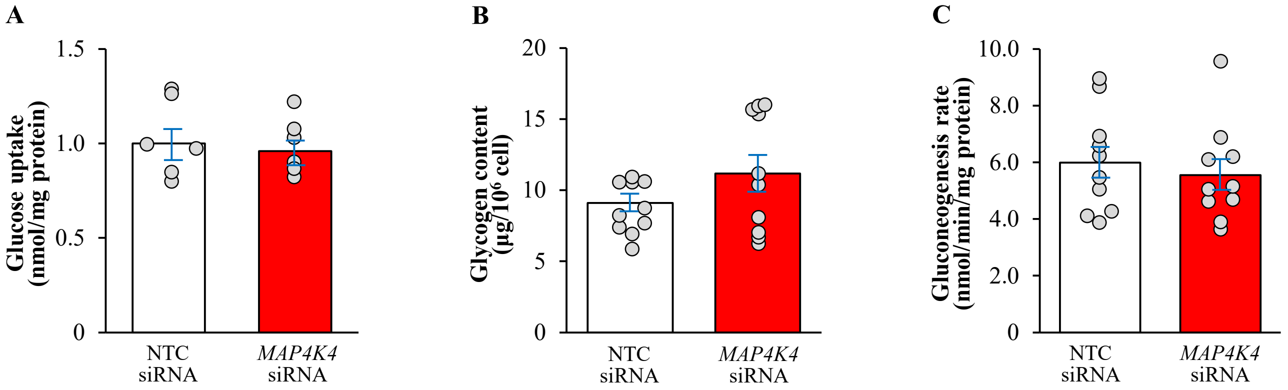

Supplementary Figure 10. Silencing of MAP4K4 in IHHs has no impact on glucose uptake (A), glycogen content (B), or gluconeogenesis rate (C). IHHs were transfected with *MAP4K4* siRNA or NTC siRNA and challenged with oleic acid for 48 hours. Glucose uptake was assessed in the absence of insulin. Data are mean ± SEM from 6-10 wells per group.

Supplementary Figure 11

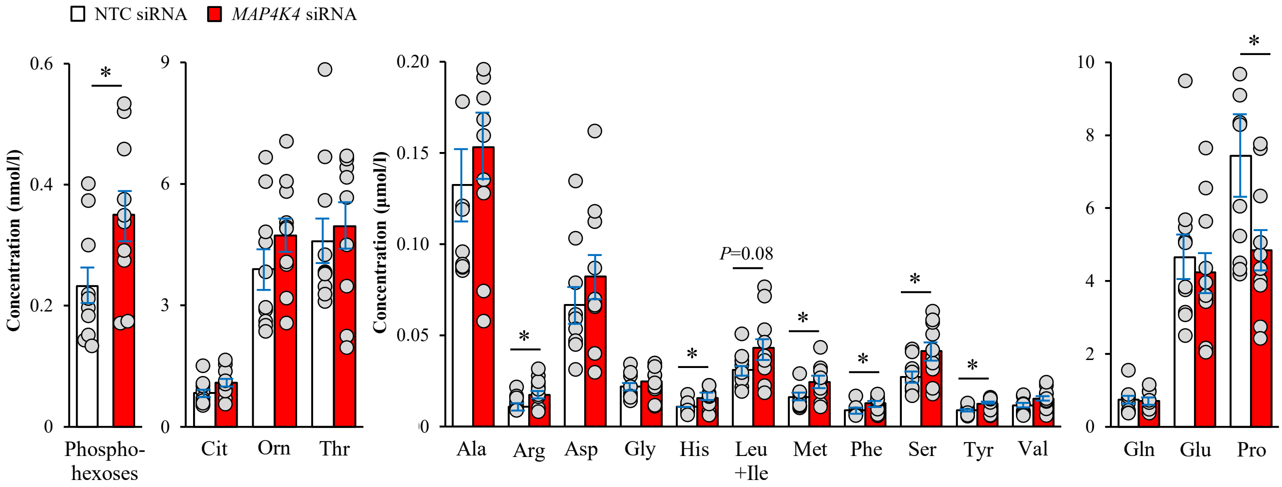

Supplementary Figure 11. Silencing of MAP4K4 in IHHs affects the concentration of phosphohexoses as well as several amino acids. IHHs were transfected with *MAP4K4* siRNA or NTC siRNA and challenged with oleic acid for 48 hours. Targeted metabolomics was carried out analyzing phosphohexoses and amino acids by multiple reaction monitoring scan. Asn, Cys, Lys, and Trp were below the level of quantification. Data are mean ± SEM from 9-10 wells per group. *$P<0.05$

Supplementary Figure 12

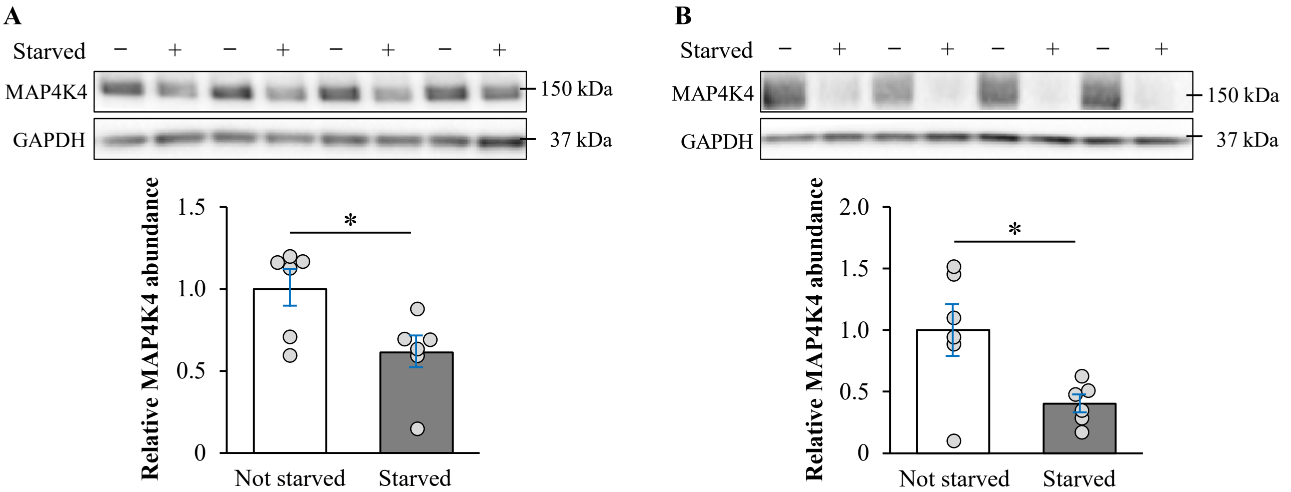

Supplementary Figure 12. The protein abundance of MAP4K4 in hepatocytes is decreased after starvation. IHHs (A) and HepG2 cells (B) were grown under basal culture conditions or in a serum-free medium for 48 hours. Cell lysates were assessed by Western blot using antibodies for MAP4K4. Densitometric analysis of protein levels and representative Western blots are presented (GAPDH used as a loading control). Data are mean ± SEM from 6 wells per group. *P<0.05

Supplementary Figure 13

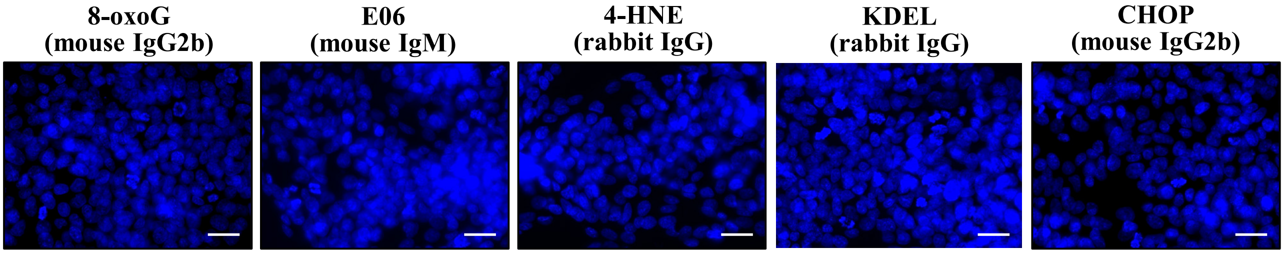

Supplementary Figure 13. Representative negative control images for immunofluorescence analysis in IHHs by substituting a primary antibody by an equivalent concentration of the corresponding Ig isotype; nuclei stained with DAPI (blue). The scale bars represent 25 μm.
